# 影像引导射频消融治疗肺部肿瘤专家共识（2015年版）

**DOI:** 10.3779/j.issn.1009-3419.2015.05.01

**Published:** 2015-05-20

**Authors:** 宝东 刘

**Affiliations:** 100053 北京，首都医科大学宣武医院胸外科 Department of Thoracic Surgery, Xuanwu Hospital, Capital medical University, Beijing 100053, China

## 1 概述

肺癌是最常见的恶性肿瘤之一。据世界卫生组织（World Health Organization, WHO）下属的国际癌症研究机构（International Agency for Research on Cancer, IARC）出版的GLOBOCAN 2012估计：全世界肺癌新发病例180万，死亡病例160万^[1]^。在中国，1988年-2005年10个肿瘤登记处18年发病死亡数据分析，肺癌发病率呈现逐年上升趋势，年平均增长1.63%，其中男性为1.30%，女性为2.34%（*P* < 0.05）^[2]^。与30年前相比，我国肺癌死亡率上升了465%，每年大约有60万人死于肺癌^[2, 3]^。

外科手术仍是治疗早期肺癌的首选，但是临床上只有20%-30%的肺癌患者适合手术治疗。随着人口老龄化，中老年肺癌的比例逐年增加，这些患者往往存在着合并症，不适合或不能耐受常规手术切除，于是许多新的局部治疗方法应运而生，如肿瘤微创消融等^[4]^（表 1）。肿瘤微创消融是指在局麻下，以肿瘤为靶心最大限度地灭活靶区的肿瘤细胞及周围0.5 cm-1 cm的正常组织，又最大限度地保护正常肺组织；具有微创、安全、适形、并发症少、操作简单、患者恢复快、效果可靠、可以重复进行等优点，现已成为一种有前途的肿瘤第四大治疗手段。目前，国内外常用的肺部肿瘤微创消融包括射频消融（radiofrequency ablation, RFA）、冷冻消融、微波消融等，但是被美国国家综合癌症网络（National Comprehensive Cancer Network, NCCN）非小细胞肺癌（non-small cell lung cancer, NSCLC）临床指引列入的只有RFA。

**1:** 肺癌外科手术排除标准 Exclusion criteria for lobectomy for primary lung cancer

主要标准（至少1项） FEV_1_占预计值≤50% DLCO占预计值≤50%
次要标准（至少2项） 年龄≥75岁 FEV_1_占预计值51%-60% DLCO占预计值51%-60% 心脏超声或右心导管估计肺动脉压>40 mmHg 左心室射血分数≤40% 休息或运动时PO_2_≤55 mmHg或SPO_2_≤88% PCO_2_>45 mmHg
FEV1：第一秒用力肺活量；DLCO：一氧化碳弥散量；PO_2_：动脉血氧分压；SPO_2_：末梢血氧饱和度；PCO_2_：动脉血二氧化碳分压。

RFA的原理是应用频率 < 30 MHz（通常在460 kHz-480 kHz之间）的交变高频电流使肿瘤组织内离子发生高速震荡，互相摩擦，将射频能转化为热能，局部温度达到60 ℃-100 ℃时，肿瘤细胞发生凝固性坏死。2000年Dupuy等^[5]^报道3例经皮RFA治疗肺部肿瘤患者，拉开了RFA应用于肺癌临床的序幕^[6-13]^。RFA的治疗效果取决于局部射频消融产生的热量传导与循环血液及细胞外液间的热对流^[14]^。但是由于肺部肿瘤具有：①血运丰富；②含气器官；③呼吸运动等特点，因此在射频消融时可能由于热沉降（heat sinking）和高阻抗（阻抗平均509 Ω±197 Ω）等效应而消融不彻底，局部复发率高^[15-17]^；容易发生气胸等并发症；疗效评价等与其他实质性器官的评价不同，比如更关注消融后肿瘤周围的磨玻璃样阴影（ground-glass opacity, GGO）等。因此射频消融治疗肺部肿瘤虽然是一种微创消融治疗技术，但是也存在潜在的风险，甚至有可能发生危及患者生命的严重并发症。由中华医学会胸心血管外科学会肺癌学组组织，首都医科大学宣武医院胸外科刘宝东起草，支修益组织国内相关专家在2014年10月-2014年11月通过讨论、电子邮件等形式，反复征求参审专家的意见，达成了影像引导射频消融治疗肺部肿瘤的专家共识，旨在规范操作技术、进行疗效评估、减少并发症和提高治疗效果。

## 2 操作平台

### 2.1 X线透视

适合周围型病灶。X线透视引导简便经济，能动态观察到进针的路径、针尖是否处于病灶中心，耗时短，但定位不够准确，不能清楚显示病灶周围血管和器官的情况，目前已逐渐被计算机横断层扫描（computed tomography, CT）所取代。

### 2.2 B超

适合贴近胸壁且瘤体较大的病灶。B超引导可实时监测，操作时间短，但它显示的病灶和穿刺位置没有CT那样直观清楚，且只能用于贴近胸壁的病灶。

### 2.3 CT

CT密度分辨率高，能显示病灶横断面位置，清楚显示心脏、大血管与病灶的关系，可以避免穿刺到血管、肺裂、肺大疱和中心坏死区。它具有定位精确、及时发现并发症和评估疗效的优点。但是不能实时监测穿刺过程，只能提供静态的横截面图像，需要反复扫描。

### 2.4 其他新技术

如C臂CT技术^[18]^、正电子发射计算机断层扫描（positron emission tomography, PET）或PET-CT。

## 3 射频电极针

### 3.1 单极射频电极针

有1个活性电极，同时拥有1个或几个回路电极板。包括多针伸展型、冷循环型和灌注型等不同的设计。

#### 3.1.1 多针伸展型射频电极针

从一个较粗的套管针内伸出阵列排列的多个电极针。是由弹性良好的多个电极针置于14 G-19 G套管针内制成的同轴电极，导入肿瘤组织后，通过针柄上的推进装置，将电极针推出针管，展开阵列排成，从而扩大了消融范围；多针伸展型射频电极针完全释放直径达3.5 cm，能产生3 cm-5 cm的坏死区，5 cm-6 cm的损伤区。

#### 3.1.2 冷循环型射频电极针

采用中空双腔设计，采用内冷却，通过电动压力泵循环冷却水至针尖，对活性电极针进行冷却，防止针尖附近组织干燥和炭化，从而降低阻抗，产生更大、更有效的凝固坏死灶。冷循环型射频电极针可分为单束型及三针集束型，后者较前者单点消融体积大。

#### 3.1.3 灌注型射频电极针

射频电极针的尖端有小孔，可通过小孔向消融组织内注射液体（常为生理盐水），提高组织电导性和热传导性，增大消融体积，防止组织炭化。

### 3.2 双极射频电极针

由2根电极针组成（分别为活性电极和回路电极），或在1根电极针的尖端同时具有活性电极和回路电极，无需回路电极板。体内有金属植入物及心脏起搏器的患者宜选用双极射频电极针。

三种类型的射频电极针在肺部肿瘤的射频消融中都可以使用，考虑到患者存在自主呼吸，肺活动度较大，建议选择多针伸展型电极针以便出针后覆盖肿瘤，减少射频电极针移动对肺的副损伤。而对邻近心脏大血管或气管支气管等重要结构的肿瘤射频消融时选择与之平行的单针（非多针伸展型）穿刺比较安全。

## 4 适应证和禁忌证

### 4.1 适应证

#### 4.1.1 根治性消融（curative ablation）

是指通过射频消融术的治疗，能够使肺部肿瘤病灶组织完全坏死，并有可能达到治愈和延长生存的目的。

##### 4.1.1.1 原发性肺癌

周围型早期NSCLC（肿瘤最大径≤3 cm，无淋巴结转移及远处转移），因心肺功能差、高龄或拒绝手术的。

##### 4.1.1.2 肺转移瘤

原发病变得到有效控制者，同时单侧肺部转移瘤总数≤3个，双侧肺转移瘤总数≤5个，肿瘤最大径≤3 cm。

#### 4.1.2 姑息性消融（palliative ablation）

是指通过射频消融术治疗，最大限度地诱导肿瘤凝固性坏死，达到减轻肿瘤负荷、缓解症状的目的。

##### 4.1.2.1 原发性肺癌

肿瘤最大径>3 cm，进行多针、多点或多次治疗，或联合其他治疗方法。

###### 4.1.2.1.1

原发性肺癌术后肺内孤立性复发。

###### 4.1.2.1.2

周围型肺癌放化疗或分子靶向药物治疗后肺部肿瘤进展或者复发。

###### 4.1.2.1.3

周围型小细胞肺癌经过放化疗以后肿瘤进展或者复发。

###### 4.1.2.1.4

合并恶性胸腔积液的周围型肺癌在胸膜活检固定以后。

###### 4.1.2.1.5

中晚期中心型NSCLC。

###### 4.1.2.1.6

肿瘤侵犯肋骨或胸椎椎体引起的难治性疼痛，对肿瘤局部骨侵犯处进行消融，可达到止痛效果。

##### 4.1.2.2 肺转移瘤

数量和大小超过根治性消融限制者。

### 4.2 禁忌证

#### 4.2.1 绝对禁忌证

有严重出血倾向、血小板 < 50×10^9^/L和凝血功能严重紊乱者（凝血酶原时间>18 s，凝血酶原活动度 < 40%）。抗凝治疗和/或抗血小板药物应在消融前至少停用5 d-7 d。

#### 4.2.2 相对禁忌证

① 有广泛肺外转移者，预期生存 < 3个月；②有严重合并症、感染期、免疫功能低下、肾功能不全者；③心脏起搏器植入、金属物植入者；④对碘对比剂过敏，无法通过增强CT扫描评价疗效；⑤美国东部肿瘤协作组（Eastern Collaborative Oncology Group, ECOG）体力状态评分>2分（表 2）。

**2:** ECOG/Zubrod体力状态评分标准 ECOG/Zubrod performance status scale

活动状态	描述
0	无症状，完全主动活动，及能够进行无限制的患病前活动
1	有症状，完全能行走，但重体力活动受限
2	有症状，能行走，生活可自理，清醒时间即白天卧床时间 < 50%
3	有症状，部分生活自理，清醒时间卧床>50%，但尚未卧床不起
4	完全失去功能，生活完全不能自理，卧床不起
ECOG：美国东部肿瘤协作组。	

## 5 检查与分期

### 5.1 术前检查

#### 5.1.1 常规检查

患者需在2周内接受血、尿、大便常规，肝、肾功能，凝血功能，肿瘤标志物，血型检查和感染筛查，心电图、肺功能等检查。

#### 5.1.2 影像检查

患者需在2周-4周内行胸部增强CT、肺部代谢显像PET或PET-CT、腹部B超、骨扫描、头颅磁共振检查。

#### 5.1.3 病理检查

经皮肺穿刺活检或者纤维支气管镜活检。

### 5.2 临床分期

见表 3，表 4。

**3:** NSCLC的TNM分期标准 Stage criteria in the TNM staging system for NSCLC

原发肿瘤（T）	
T_X_	原发肿瘤无法评估，或虽然痰或气管灌洗液中发现恶性细胞，但影像学或支气管镜检查未发现肿瘤
T0	无原发肿瘤证据
Tis	原位癌
T1	肿瘤最大径≤3 cm，由肺组织或脏层胸膜包绕，支气管镜下未见病变侵及叶支气管
T1a	肿瘤最大径≤2 cm
T1b	肿瘤最大径>2 cm，但≤3 cm
T2	肿瘤最大径>3 cm但≤7 cm，或具有以下任一特征（具有这些特征的T2肿瘤如果≤5 cm则归为T2a）：累及主支气管，但距隆突≥2 cm；累及脏层胸膜；伴随延伸到肺门区的肺不张或阻塞性肺炎，但没有累及全肺
T2a	肿瘤最大径>3 cm，但≤5 cm
T2b	肿瘤最大径>5 cm，但≤7 cm
T3	肿瘤最大径>7 cm或直接侵及以下部位：胸壁（包括肺上沟瘤）、膈肌、膈神经、纵隔胸膜、壁层心包；或肿瘤位于主支气管内距隆突 < 2 cm，但又未累及隆突；或伴随全肺不张或阻塞性肺炎；或在同一肺叶内有其他转移结节
T4	任何大小肿瘤侵及到以下部位：纵隔、心脏、大血管、气管、喉返神经、食管、椎体、隆突；或在同侧不同肺叶内有其他转移结节
区域淋巴结（N）	
N_X_	区域淋巴结无法评估
N0	无区域淋巴结转移
N1	同侧气管周围和/或同侧肺门淋巴结及肺内淋巴结转移，包括原发病灶直接侵及所致
N2	同侧纵隔淋巴结和/或隆突下淋巴结转移
N3	对侧纵隔，对侧肺门，同侧或对侧斜角肌，或锁骨上淋巴结转移
远处转移（M）	
M_X_	远处转移不能评估
M0	无远处转移
M1	有远处转移
M1a	对侧肺叶内有转移结节；伴有胸膜结节或出现恶性胸膜或心包积液
M1b	肺外远处转移
第7版分类更新的内容用红色字体表示。NSCLC：非小细胞肺癌；TNM：肿瘤-淋巴结-转移。	

**4:** 第7版NSCLC的TNM分期 IASLC lung cancer staging project: Proposed TNM categories and stage groupings for NSCLC

隐匿癌	T_X_	N_0_	M_0_
0期	Tis	N_0_	M_0_
ⅠA期	T_1a, b_	N_0_	M_0_
ⅠB期	T_2a_	N_0_	M_0_
ⅡA期	T_1a, b_	N_1_	M_0_
	T_2a_	N_1_	M_0_
	T_2b_	N_0_	M_0_
ⅡB期	T_2b_	N_1_	M_0_
	T_3_	N_0_	M_0_
ⅢA期	T_1, 2_	N_2_	M_0_
	T_3_	N_1, 2_	M_0_
	T_4_	N_0, 1_	M_0_
ⅢB期	T_4_	N_2_	M_0_
	T_1-4_	N_3_	M_0_
Ⅳ期	任何T	任何N	M_1a, b_
第7版分期更新的内容用红色字体表示。IASLC：国际肺癌研究协会。			

## 6 术前准备

### 6.1 制定计划

根据CT或PET-CT描述肿瘤的位置、大小、数目、形状，以及与心脏大血管、气管支气管等的关系，确定体位和进针路径。

### 6.2 仪器设备

CT、射频消融发生器、射频电极针、胸穿或胸腔闭式引流包、心电监护仪、吸氧装置、抢救车等相关设备。

### 6.3 药品准备

准备用于麻醉、镇痛、镇咳、止血、扩冠、降压等药物。

### 6.4 患者准备

① 患者及家属（被委托人）签署知情同意书；②术前4 h禁食；③必要时备皮；④必要时建立静脉通道；⑤必要时术前口服镇咳剂；⑥术前教育。

## 7 操作步骤

### 7.1 体位

根据病变部位患者采用不同的体位，原则上是穿刺距离最短和比较舒适的体位。

### 7.2 消毒与麻醉

碘酒、酒精消毒，铺无菌巾；穿刺点处用1%~2%利多卡因局部浸润麻醉，直至胸膜。对于儿童、术中不能配合、预计手术时间长、肿瘤贴近壁层胸膜可能引起剧痛的患者，推荐采用清醒镇静或全身麻醉。麻醉前评估可参照美国麻醉医师协会（American Society of Anesthesiologists, ASA）的分级标准（表 5），≤Ⅲ级的患者方可进行RFA治疗。

**5:** ASA麻醉风险分级 ASA classification of anesthesia risk

分级	标准
Ⅰ级	体格健康，发育营养良好，各器官功能正常
Ⅱ级	除外科疾病外，有轻度合并疾病，功能代偿健全
Ⅲ级	合并疾病较严重，体力活动受限，但尚能应付日常活动
Ⅳ级	合并疾病严重，丧失日常活动能力，经常面临生命威胁
Ⅴ级	无论手术与否，生命难以维持24 h的濒死患者
ASA：美国麻醉医师协会。	

### 7.3 定位与穿刺

CT是最常用和最准确的影像引导方式之一，操作过程是将射频电极在CT引导下通过穿刺点穿刺入靶肿瘤中。每次CT扫描的范围包括靶肿瘤即可。通过三维重建CT影像确认射频电极处于正确位置后，进行消融。

### 7.4 消融

根据射频消融治疗仪的类型、射频电极针的型号、肿瘤大小及其与周围组织结构的关系设置治疗参数（肺部肿瘤射频消融可以根据不同设备生产商推荐的参数进行适当调整）。为确保靶肿瘤完全消融，消融范围应包括靶肿瘤及瘤周0.5 cm-1.0 cm肺组织的所谓“消融区”。消融结束，拔出射频电极前要消融穿刺针道，以减少肿瘤种植和出血。消融过程需要监测心率、血压和血氧饱和度，同时要观察患者的呼吸、疼痛、咳嗽、咯血等情况，必要时对症处理。

#### 7.4.1 小肿瘤

肿瘤≤3个、直径≤3 cm者，单次RFA治疗。

#### 7.4.2 中肿瘤

直径3 cm-5 cm的肿瘤，单次多点RFA治疗。

#### 7.4.3 大肿瘤

直径>5 cm的肿瘤，单次多点RFA治疗，随后放疗或多次多点RFA治疗。

#### 7.4.4 特殊部位肿瘤

如邻近心脏大血管、气管支气管、食管、膈肌和胸膜顶病灶，建议使用单针，穿刺方向尽可能与重要结构平行，并保持0.5 cm以上。

### 7.5 术后扫描

立即进行再次CT全胸腔扫描，评价技术是否成功（肿瘤是否按照消融程序完成治疗和覆盖完全），同时观察是否有并发症的发生。

### 7.6 术后处理

术后平卧2 h-4 h，并监测生命体征。24 h-48 h后拍胸片或CT扫描，观察是否有并发症的发生（如无症状性气胸或胸腔积液）。

## 8 并发症及处理

射频消融术是一种相对安全的局部治疗手段，其并发症分级参照美国介入放射学学会（Society of Interventional Radiology, SIR）影像引导肿瘤消融国际工作组（International Working Group on Image-Guided Tumor Ablation）的标准^[19]^（表 6）。按照发生时间分为即刻并发症（RFA后≤24 h）、围手术期并发症（RFA后24 h-30 d）及迟发并发症（RFA后>30 d）。

**6:** SIR并发症的定义与分级 SIR definitions and grading system of complication

并发症分类	定义
副反应	疼痛
	消融后综合症
	无症状胸腔积液
	无后果的邻近结构损伤
轻微	没有不良结果，不需要治疗
	没有不良结果，仅是名义上的治疗，包括仅需要过夜观察
严重	需要住院进行小的治疗 < 48 h
	需要留院进行大的治疗>48 h，提升护理级别，延长住院时间
	导致永久不良后遗症
	死亡
SIR：美国介入放射学学会。	

射频消融治疗肺部肿瘤的并发症分两种：穿刺相关并发症如肺内出血、血胸、气胸、心包填塞、空气栓塞等；消融相关并发症如胸痛、胸膜反应、咳嗽、皮肤灼伤等。肺部肿瘤射频消融的死亡率为0-5.6%^[20]^。在样本量大于100例的文献中，射频消融的死亡率为0-2.2%，严重并发症和轻微并发症发生率分别3%-24.5%和21.3%-64.9%，其死亡原因有出血、肺炎、肺间质纤维化恶化、肺栓塞、急性心衰、呼吸衰竭等^[21]^。

### 8.1 疼痛

推荐美国国家癌症研究所的常见毒性标准（common toxicity criteria, CTC version 2.0）报告：0级，没有疼痛；1级，轻度疼痛，不影响功能；2级，中度疼痛，需要止痛药，干扰功能但不干扰日常活动；3级，严重疼痛，需要止痛药，严重影响日常生活活动；4级，伤残性疼痛。

#### 8.1.1 术中疼痛

① 原因：在局麻条件下手术，一般均有不同程度的疼痛，可能是热传导刺激胸膜神经所致。Okuma等^[22]^单变量和多变量分析研究认为，疼痛的发生与病变距离胸壁在1 cm以内显著相关。②治疗：如果疼痛剧烈，需要对胸膜彻底麻醉；或者需要镇痛剂，甚至清醒镇静麻醉；或者降低靶温度到70 ℃，几分钟后，再逐渐升高靶温度；或者通过三维重建CT图像，观察有无射频针接近胸膜，可以旋转射频针，再消融；或者向胸腔内推射频针，使脏层胸膜离开壁层胸膜，即造成人工气胸^[23, 24]^。

#### 8.1.2 术后疼痛

一般为1级-2级疼痛，可持续数天，也有人持续1周-2周，一般无需特别处理，很少出现中度以上的疼痛，可以用非甾体类药物止痛。

### 8.2 消融后综合征

约2/3患者可能发生。①原因：肿瘤坏死吸收，其严重程度及持续时间取决于产生坏死的体积以及患者的总体情况，小的病灶不太可能出现消融后综合征，大部分患者症状持续2 d-7 d，消融肿瘤体积较大者则持续2周-3周。②治疗：大多数一过性自限性症状，对症支持即可。少数病人需要给予非甾体类药物，必要时可以适量短时应用小剂量糖皮质激素。

### 8.3 气胸

发生率为5%-63%^[20, 22]^。推荐CTC version 2.0报告：0级，没有气胸；1级，不需要干预；2级，需要放置胸腔闭式引流；3级，需要胸膜固定或手术治疗；4级，威胁生命。

#### 8.3.1 术中气胸

① 原因：Hiraki等^[25]^报道发生气胸的危险因素包括男性（肺活量大）、无胸部手术史（没有胸膜粘连）、消融多个肿瘤（多次穿刺）、中下叶病变（肺活动度大）、病变小且深在（难以定位，需反复穿刺）、大肿瘤（多点消融，反复穿刺、集束针、消融时间超过3 h）有关。Sano等^[26]^研究结果表明高龄、多头伸展针和大功率输出是气胸发生有统计学意义的危险因素。②治疗：少量气胸可不予处置，中等至大量气胸可胸穿抽气或放置胸腔闭式引流装置。有文献^[20]^报道3.3%-38.9%平均11%需要放置胸腔闭式引流，还有文献^[25]^报道多见于上肺叶肿瘤射频消融，可能的原因是上叶肺泡胸膜压力梯度高，患者直立时，大量气体持续进入胸腔。③预防：为减少气胸的发生，关键在于穿刺技术要熟练，进针速度快和穿刺准确避免多次穿刺十分重要，同时建议使用同轴系统射频针，经注水孔注射生理盐水或麻醉剂于胸膜连接处，使肺外组织增厚。

#### 8.3.2 迟发性气胸

发生率约10%。一般认为消融后72 h发生的气胸称为迟发性气胸，处理同前。

### 8.4 胸腔积液

消融后经常可以见到少量胸腔积液，发生率1.3%-60%（13.4%）^[20]^。推荐CTC version 2.0报告：0级，没有胸腔积液；1级，无症状和不需要干预；2级，有症状，需要利尿；3级，有症状，需要吸氧或胸腔穿刺；4级，威胁生命（需要气管插管）。①原因：与消融过程中高温胸膜受刺激有关。导致胸腔积液发生的危险因素有：合并慢阻肺、大病灶、一次消融多个病灶、病灶靠近胸膜（< 10 mm）、消融时间长等^[25]^。②治疗：一般观察或保守处理即可。如果出现中到大量胸腔积液，需要行穿刺抽吸或胸腔闭式引流，需要胸腔引流者低于10%。③预防：消融时尽量远离胸膜。

### 8.5 出血^[20]^

术中咯血发生率为3.3%-18.2%（11.1%），大咯血的发生率极低。肺内出血发生率为0-11%（7.1%），与咯血和术后血痰并不一致。血胸发生率为1.9%-16.7%（4.3%）。①原因：没有发现特殊的高危因素^[22]^。但也有人认为与病灶小、穿刺路径长、合并慢阻肺、肺动脉高压有关。②治疗：术中出现咯血后立即消融，同时静脉输注止血药，咯血逐渐停止或减少。肺内出血可自动吸收。术后血痰多具有自限性，可持续3 d-5 d。如果术中发现少量胸腔积液，可以密切观察，保守治疗；如果出现中到大量胸腔积液，说明有活动出血，需要行穿刺抽吸或胸腔闭式引流，文献报道约10%左右需要行胸腔引流，同时应用止血药物。血胸保守治疗无效者，可行介入栓塞治疗或剖胸探查。③预防：由于消融本身可以使血液凝固，随着消融治疗的进行出血会逐渐停止，故在具体消融治疗过程中大出血的发生率并不高。穿刺时避开血管走行区或者不张的肺组织等。术前要注意血小板计数、凝血时间和抗凝药的应用等。

### 8.6 咳嗽

推荐CTC version 2.0报告：0级，没有咳嗽；1级，不需要干预可以缓解；2级，需要止咳药缓解；3级，严重咳嗽或痉挛性咳嗽，对治疗无效。①原因：术中剧烈咳嗽可能与病灶局部温度增高刺激肺泡、支气管内膜或胸膜所致。术后咳嗽是射频消融局部肿瘤组织坏死及其周围肺组织热损伤引起的炎症反应所致。②治疗：口服镇咳剂或经过射频针注水孔注入利多卡因即可缓解，部分患者可能只有在消融结束后咳嗽停止。术后咳嗽可适当给予止咳化痰药。③预防：前半小时口服可待因可减轻咳嗽反应。

### 8.7 胸膜反应

① 原因：消融过程中刺激了支配壁层胸膜的迷走神经，兴奋的迷走神经可使心率减慢、甚至心跳停止。局部麻醉不充分；部分患者对疾病的不了解，对治疗手段的恐惧，甚至处于高度紧张状态。②治疗：针对这类患者，建议暂停消融，局部充分麻醉，并适当应用阿托品、镇静剂等药物。③预防：术前沟通，患者精神放松，或者彻底麻醉附近胸膜。

### 8.8 少见并发症

其他潜在致命的并发症包括支气管胸膜瘘引起的顽固性气胸、空气栓塞和肺炎。其他严重并发症包括邻近神经损伤（如臂丛、肋间、膈、喉返等神经对热敏感）、心包填塞、针道种植、肺脓肿、皮肤灼伤等。

## 9 随访及疗效评估

### 9.1 随访

复查胸部CT，肿瘤标志物等，有条件者可选择PET-CT检查。主要评价病灶是否完全消融，有无局部进展、新发病灶等。PET-CT检查判断疗效更准确，并有助于确定有无肺外转移。评价患者生活质量或姑息治疗的改善情况、生存时间等。

### 9.2 消融区影像学改变^[27, 28]^

#### 9.2.1 CT改变

术后4周-6周复查胸部增强CT，并以此为基线进行评价。术后2年内每3个月复查胸部CT，2年后每6个月复查一次。

##### 9.2.1.1 早期改变（1周内）

消融区增大，病灶内蜂窝状低密度改变，周边包绕环周或部分GGO是治疗成功的表现。但是，单纯用GGO衡量有可能高估消融效果，该区域病理学检查可见“鬼影”细胞，可能是由于热消融后肿瘤突然凝固性坏死和微循环破坏，阻止酶从细胞内溶酶体释放，以及炎性细胞浸润，延迟细胞自溶所致，需要用还原型烟酰胺腺嘌呤二核苷酸（NADH）离体活体染色及其他特殊染色进行鉴别。

##### 9.2.1.2 中期改变（1周-3个月内）

消融区持续增大，其周边由于炎症吸收可能出现环绕清晰锐利的强化环。

##### 9.2.1.3 后期改变（3个月后）

与基线相比，消融区在3个月后保持稳定或稍大，6个月后大小稳定或逐渐缩小，并可有多种演变模式（如纤维化、空洞形成、结节、肺不张、消失等），或组合出现。

#### 9.2.2 PET-CT改变

有条件者术后3个月复查PET-CT，以后每6个月复查一次。用标准摄取比（standard uptake value, SUV）描述。

### 9.3 局部疗效评估

推荐使用改良的实体瘤疗效评价标准（Response Evaluation Criteria in Solid Tumors, RECIST）。一般在射频消融后3个月评价，见表 7。

**7:** 改良实体瘤疗效评价标准 Modiﬁed RECIST criteria used to evaluate treatment response

效果	CT（大小）	CT（密度）	PET
完全消融	消失或缩小	无增强区	无代谢区
不完全消融	不变	增强区无变化	有高代谢区且无变化
局部进展	增大10 mm	增强区增大	新出现高代谢区或高代谢区增大
CT：计算机断层扫描；PET：正电子发射型计算机断层显像。			

#### 9.3.1 完全消融

CT提示出现下列表现任何一项，如靶肿瘤消失，无强化的空洞、实性结节、肺不张和纤维化等。或者PET-CT提示靶肿瘤无核素浓聚或SUV值正常。

#### 9.3.2 不完全消融

CT提示靶肿瘤空洞形成不完全，有部分实性或液性成分，且CT扫描有造影剂强化；靶肿瘤部分纤维化仍存有部分实性成分，且实性部分CT扫描有造影剂强化；靶肿瘤呈实性结节，大小无变化或增大，且伴CT扫描造影剂有强化征象。PET-CT提示靶肿瘤消融后仍有核素浓聚或SUV值仍高于正常。

#### 9.3.3 肿瘤局部进展

CT提示靶肿瘤完全消融后，瘤周又出现散在、结节状、不规则偏心强化；PET-CT提示消融后靶肿瘤无核素浓聚或SUV值正常后，又出现核素浓聚或SUV值高于正常。对局部肿瘤进展的患者需要进行二次消融或其他治疗。

### 9.4 远期疗效评估

术后随访2年-5年，使用*Kaplan*-*Meier*分析或生存数表法计算生存率（overall survival, OS）、无疾病生存（disease-free survival, DFS）等。

## 10 综合治疗

热沉降效应虽然保护血管、防止大血管出血，同时它们也是射频消融不彻底的一个主要因素。为克服这个问题，通过药物降低血流，在消融过程中特殊血管采用血管球囊临时闭塞特定血管、动脉栓塞或化疗栓塞等几种策略减少血流^[29]^。

射频消融联合其他方法进行治疗是目前肺部肿瘤研究的重要内容之一，包括射频消融与外科^[30]^、放疗^[31]^、化疗^[32]^和分子靶向药物^[33]^等的联合，可以提高肿瘤的局部控制率，延长患者的生存。

## 11 总结

最近研究表明：射频消融治疗不能手术的早期NSCLC（肿瘤直径≤3 cm）的1年、3年和5年的生存率分别达到90%、70%和50%，且死亡率小于2%。射频消融治疗肺部肿瘤具有创伤小、疗效确切、安全性高、患者恢复快、操作相对简单、适用人群广等特点。这些临床证据让我们相信未来这一技术会在肺部肿瘤的综合治疗中得到越来越广泛的应用，目前已成为继手术、放疗、化疗之后的第四大治疗模式。但还需前瞻性随机多中心临床研究对其疗效加以证明，并与其他治疗手段如手术^[34, 35]^和立体定向放射治疗^[36, 37]^进行比较研究；再次，RFA作为一种局部微创消融治疗手段，需要联合其他治疗手段提高疗效，比如放疗、全身治疗（根据基因检测结果，选择用化疗还是分子靶向药物治疗）等。

总之，从临床实践的角度看，有关射频消融技术治疗原发性肺癌的有效性和安全性都已得到临床验证，但是不同单位开展的方法并不一致，希望通过本共识达到规范化推广的目的。本共识虽然借鉴了许多国际指南和国内外的最新进展，经过多次认真讨论和反复修改，仍难免存在不足和局限性，因此，需要在以后的临床实践中不断补充，动态完善，制定出符合我国国情的射频消融技术临床指南。
